# A novel Notch1 missense mutation (C1133Y) in the Abruptex domain exhibits enhanced proliferation and invasion in oral squamous cell carcinoma

**DOI:** 10.1186/s12935-017-0496-5

**Published:** 2018-01-08

**Authors:** Yang Zheng, Zhao Wang, Xu Ding, Wei Zhang, Gang Li, Laikui Liu, Heming Wu, Wenyi Gu, Yunong Wu, Xiaomeng Song

**Affiliations:** 1 Key Laboratory of Oral Diseases of Jiangsu Province and Stomatological Institute of Nanjing Medical University, 140, Hanzhong Road, Nanjing, 210029 People’s Republic of China; 2 Department of Oral and Maxillofacial Surgery, Affiliated Stomatological Hospital, Nanjing Medical University, 136, Hanzhong Road, Gulou District, Nanjing, 210029 People’s Republic of China; 3 Department of Stomatology, Affiliated Hospital of Xuzhou Medical College, 99, Huaihai West Road, Xuzhou, 221000 People’s Republic of China; 4 Australian Institute for Bioengineering and Nanotechnology, the University of Queensland, Brisbane, QLD 4006 Australia

**Keywords:** Abruptex, Notch1, OSCC, EMT, EGFR, AKT

## Abstract

**Background:**

Notch1 has been regarded as a fundamental regulator in tissue differentiation and stem cell properties. Recently, Notch1 mutations have been reported intensively both in solid tumors and in hematopoietic malignancies. However, little is known about the biological effect and the clinical implication of these reported mutations. Previously, we discovered several missense mutations in the Notch1 receptor in a Chinese population with oral squamous cell carcinoma (OSCC).

**Methods:**

We selected a ‘hotspot’ mutation in the Abruptex domain (C1133Y). The expression of Notch1 was determined by western blot and real-time qPCR in OSCC cell lines transfected with pcDNA3.1-Notch1^WT^, pcDNA3.1-Notch1^C1133Y^, or pcDNA3.1 empty vector. CCK-8 assays were used to assess cell proliferation. Flow cytometry and western blot were used to confirm the alteration of cell cycle after transfection. Transwell assays and the detection of Epithelial-to-mesenchymal transition (EMT) markers were used to determine the invasive ability. The effects of Notch1 C1133Y mutation were analyzed by Immunofluorescence staining and the expression of EGFR-PI3K/AKT signaling.

**Results:**

We demonstrated that Notch1^C1133Y^ mutation inactivated the canonical Notch1 signaling. We identified an oncogenic phenotype of this mutation by promoting cell proliferation, invasion and by inducing EMT in OSCC cell lines. We found that the Notch1^C1133Y^ mutation exhibited a decreased S1-cleavage due to the impaired transport of Notch1 protein from the endoplasmic reticulum (ER) to the Golgi complex, which was consistent with the observation of the failure of the Notch1^C1133Y^ mutated receptor to present at the cell surface. Importantly, the mutated Notch1 activated the EGFR-PI3K/AKT signaling pathway, which has been confirmed as an overwhelming modulator in OSCC.

**Conclusions:**

Taken together, our findings revealed for the first time a novel Notch1 mutation that enhances proliferation and invasion in OSCC cell lines. The Notch1 C1133Y mutation impairs the processing of notch1 protein and the critical links between the mutated Notch1 and the activated EGFR-PI3K/AKT signaling pathway.

**Electronic supplementary material:**

The online version of this article (10.1186/s12935-017-0496-5) contains supplementary material, which is available to authorized users.

## Background

Oral squamous cell carcinoma (OSCC) is a locally aggressive epithelial neoplasm, having a propensity of lymph-node metastasis and a poor prognosis [1–3]. Although the etiology and mechanisms of OSCC malignant progression, such as tumor proliferation, invasion, metastasis and stem cell properties, are intricate and poorly understood, it has been clearly elucidated that both genetic and environmental elements play a role in OSCC progression [1, 2, 4].

Notch is a highly conserved and fundamental signaling system that mediates cell–cell interactions during animal development through highly context-dependent and cell-type-dependent effects on cell growth, fate determination and survival. Aberrations in Notch signaling or components of the signaling system underlie various human diseases including carcinogenesis [5–7]. Recent whole exome sequencing of OSCC showed substantial high rates of somatic mutations, including Notch1, in Caucasian populations, revealing a common genetic cause for malignancy [8, 9]. A multitude of mutations in Notch1 has been reported in cutaneous and lung squamous cell carcinoma in Caucasian populations, in OSCC in Japanese populations, and in OSCC in Chinese populations [10–12]. Although many Notch1 mutations have been discovered by sequencing in numerous malignancies, subsequent functional studies are lacking. As a result, the oncogenic role of specific Notch1 mutations in tumor progression is still speculative and requires further verification.

The human Notch1 receptor is synthesized as a single 300 kD polypeptide in the endoplasmic reticulum (ER), and it is cleaved by a furin convertase during post-translation in the Golgi complex into 120 and 180 kD fragments (S1 cleavage). The two fragments are then presented on the cell surface as a functional heterodimer. Multiple ligands (Jagged and Delta) can interact with the extracellular domain of the Notch1 receptor. Two additional cleavage reactions (S2 and S3) are then triggered, liberating the Notch intracellular domain (NICD). The intracellular domain then translocates into the nucleus where it interacts with the CSL DNA-binding protein (CBF1) to activate downstream target genes, particularly the members of the Hairy/Enhancer of Split family (HES) and Hairy/Enhancer-of-Split related with YRPW motif (HEY) [7, 13, 14].

Epithelial-to-mesenchymal transition (EMT) has been well-elucidated and associated with tumor invasion, metastasis and tumor survival, and EMT is frequently observed in OSCC [15–17]. EMT is a crucial early event in tumor progression and is characterized by the down-regulation of epithelial markers (e.g., Beta-catenin and E-cadherin) and the up-regulation of mesenchymal markers (e.g., N-cadherin and Vimentin) [18]. The EMT process endows epithelial cells with mesenchymal cell properties, reduces intercellular adhesion, and increases the capacity for invasion [19]. In addition, Notch1 plays as a fundamental regulator in the induction as well as the maintenance of EMT and tumor progression [20, 21].

Epithelial growth factor receptor (EGFR) is composed of an extracellular ligand binding domain, transmembrane segment, and cytoplasmic domain with tyrosine kinase activity [22]. The binding of a ligand to the EGFR triggers EGFR autophosphorylation and downstream signaling transduction cascades, including MAPK, STAT3, and PI3K/AKT pathways. This process is of high biological and clinical significance because increased EGFR levels have been observed in ∼ 90% of the OSCC and believed to be an early event in OSCC pathogenesis [23]. Crosstalk between Notch1 and EGFR signaling in cell proliferation and cancer expression has been observed in genomics and can be either synergistic or antagonistic depending on the different contexts [24–26]. Notch1-dependent regulation of EGFR has been described in several types of cancers.

In our previous study, we examined Notch1 mutational status in OSCC in Chinese patients and observed a mutation rate of 43% in the patient population with a poorer clinical outcome [12]. We also identified the spectrum of Notch1 mutations. One of the common domains with mutations is the Abruptex domain (amino acids 907–1143) in the EGF-like repeats (EGF-like repeats 24–29), which has also been observed by other researchers [8, 9]. The Abruptex domain contains the most mutations (13 or 31%), including three nonsense mutations and a hotspot mutation (C1133Y). Because the Abruptex domain plays a role in suppressing cis-inhibition of Notch1 signaling, mutations in this region are thought to be gain-of-function [27, 28].

In this study, we established full-length wild-type Notch1 (Notch1^WT^) and the Abruptex domain hotspot mutant Notch1 (Notch1^C1133Y^) vectors and discovered the functional effects of the mutation on Notch1 protein maturation and transportation. Results showed that compared with OSCC cell lines transfected with Notch1^WT^, cells transfected with Notch1^C1133Y^ exhibited increased cell proliferative and invasive property. We also discovered an EMT-like phenotype in cells transfected with Notch1^C1133Y^. Importantly, the Notch1^C1133Y^ activated the EGFR-PI3K/AKT signaling pathway, which has been confirmed as an overwhelming modulator during oral carcinogenesis.

## Methods

### Cell culture, plasmid construction, and transfection experiments

CAL27 cell line was purchased from the American Type Culture Collection (ATCC), and the HN4, HN6, and HN13 cell lines were obtained from the Shanghai Ninth People’s Hospital (Shanghai, China). All cells were cultured in Ham’s F12 medium and Dulbecco’s Modified Eagle’s medium supplemented with 10% fetal bovine serum (FBS) and 100 units/ml penicillin/streptomycin (Invitrogen) in humidified incubators at 37 °C in an atmosphere of 5% CO_2_. The wild-type Notch1 or mutant Notch1 vectors containing full-length wild-type Notch1 (Notch1^WT^) or mutant Notch1 (Notch1^C1133Y^) cDNA inserted into pcDNA3.1 were synthesized and constructed by Generay Biotech (Shanghai, China). For transfection, cells (5 × 10^5^ cells per well in 6-well plates) were cultured to 50% confluence in complete growth medium, and the medium was then replaced with serum-free medium for 12–16 h. The purified pcDNA3.1, pcDNA3.1-Notch1^WT^ and pcDNA3.1-Notch1^C1133Y^ plasmids were transfected into cells using Lipofectamine 2000 (Invitrogen) according to the manufacturer’s instructions. Forty-eight hours after transfection, the medium was supplemented with 400 μg/ml G418 (Invitrogen). Two weeks later, successful transfection was confirmed by evaluating Notch1 mRNA by real-time qPCR (> 20-fold less than controls) and by western blot analysis using anti-Notch1 antibodies (see below).

### Western blot analysis

Total protein was lysed using lysis buffer (Beyotime, China) containing phosphatase inhibitor and protease inhibitor cocktails. Coomassie Brilliant Blue was utilized to quantify the protein lysates, and bovine serum albumin (BSA) was used as the standard. All proteins (10 μg) were separated using SDS–polyacrylamide gels and transferred onto polyvinylidene fluoride (PVDF) membranes (Millipore), which were then blocked with 5% BSA at room temperature for 2 h and hybridized with primary antibodies (diluted 1:1000) specific for Notch1 (clone D1E11), cleaved-NICD (clone D3B8), CDK2, CDK4, cyclin D1, cyclin D3, P21, P27, EGFR, p-EGFR, AKT, p-AKT, PI3K, p-Stat5, p-Shc, p-Gab1 (purchased from CST); HES-1 (purchased from Abcam); Beta-actin, Beta-catenin, E-cadherin, Vimentin, N-cadherin, HES-2 and SNAI1, SNAI2 (purchased from Bioworld) overnight at 4 °C followed by incubation with anti-goat IgG HRP-conjugated secondary antibodies (Zhongshan Goldenbridge Bio) for 1 h at room temperature. Immunoreactive bands were detected using an Immobilon Western Chemiluminescent HRP Substrate (Millipore) and visualized using the ImageQuantLAS4000 mini imaging system (General Electric). Protein expression levels were counted as gray values relative to Beta-actin according to the analyses of the bands using ImageJ software. Three independent experiments were analyzed for quantification.

### Real-time qPCR

According to the manufacturer’s protocol, total RNA was extracted from cells using TRIzol reagent (Invitrogen) and was converted to cDNA using 5 × PrimeScript RT Master Mix (TaKaRa) at 37 °C for 15 min and 85 °C for 5 s. Primer sequences were obtained from the Primer Bank and were used in quantitative real-time PCR (RT-qPCR) with a 7900HT Real-Time PCR System (Applied Biosystems). The delta delta Ct method for quantitation of relative gene expression was used to determine the mean expression of each target gene normalized to the geometric mean of GAPDH. All primers were designed and synthesized to target the specific sequences of the genes as follows:

Notch1: F: 5′-AGCAAGTTCTGAGAGCCAGG-3′
  R: 5′-TAACAGGCAGGTGATGCTGG-3′
GAPDH: F: 5′-GAAGGTGAAGGTCGGAGTC-3′
  R: 5′-GAGATGGTGATGGGATTTC-3′
HES-1: F: 5′-TCAACACGACACCGGATAAAC-3′
  R: 5′-GCCGCGAGCTATCTTTCTTCA-3′
HES-2: F: 5′-CCAACTGCTCGAAGCTAGAGA-3′
  R: 5′-AGCGCACGGTCATTTCCAG-3′
SNAI1: F: 5′-TCGGAAGCCTAACTACAGCGA-3′
  R: 5′-AGATGAGCATTGGCAGCGAG-3′
SNAI2: F: 5′-TGTGACAAGGAATATGTGAGCC-3′
  R: 5′-TGAGCCCTCAGATTTGACCTG-3′

### Immunofluorescence staining

HN6 cells (2 × 10^4^) expressing Notch1^WT^, Notch1^C1133Y^, or control vector were seeded onto sterile glass coverslips in 24-well plates at approximately 20% confluence. After treatment for 24 h, cells were fixed in 4% paraformaldehyde and permeabilized in 1% Triton. After incubation overnight with rabbit anti-Notch1 antibody (1:100, D6F11, CST, USA), mouse anti-Calnexin antibody (1:500, Abcam), and mouse anti-CM130 antibody (1:500, Abcam), cells were stained for 1 h with goat anti-mouse IgG antibody Cy3 (1:500, Abcam) or goat anti-rabbit IgG antibody FITC (1:500, Abcam) and counterstained with DAPI (Sigma, St. Louis, MO). Cells were subsequently viewed by a fluorescence microscopy (ZEISS, Germany).

### Flow cytometry

For cell-cycle analysis, stable cells were harvested and washed in phosphate-buffered saline (PBS) and fixed in 75% ice-cold ethanol for 30 min at 4 °C. Cells were then washed twice in PBS, stained with propidium iodide (50 μg/ml) in the presence of 50 μg/ml RNase A (Sigma-Aldrich) and incubated for 1 h at room temperature. The cell-cycle analysis was performed on a FACSCalibur flow cytometer (BD Biosciences) and CellQuest Pro software (BD Biosciences). Flow cytometric analysis of apoptotic cells was performed by staining the cells using the Annexin V Apoptosis Detection Kit (BD Pharmingen) according to the manufacturer’s protocol. The percentages of cells in specific cell-cycle stages in Notch1^C1133Y^ cells were compared with those in Notch1^WT^ cells.

### Transwell invasion assays

According to the manufacturer’s instructions, Transwell chambers (8 μm pore size; Millipore) were used to detect cell invasive ability. A total of 3 × 10^4^ cells were seeded into the upper chamber of each insert and incubated at 37 °C for 24 h. Similar inserts coated with Matrigel (BD Biosciences) were used to determine invasive potential in cell invasion assays. Chambers were fixed in 4% paraformaldehyde for 30 min and then dyed with crystal violet substrate. The non-invaded cells on the upper chamber surface were removed, and the invaded cells on the surface were subsequently viewed and counted by a microscopy (ZEISS, Germany).

### CCK-8 proliferation assays

Cells were seeded into 96-well microplates at a density of 2 × 10^3^ cells per well and incubated in fresh medium containing 10% CCK-8 reaction solution. After incubation for 1 h, the absorbance was measured on a microplate spectrophotometer (Multiskan MK3, Thermo) at a wave length of 450 nm according to the manufacturer’s instructions. Five independent experiments were performed. The growth curves were illustrated using Graphpad Prism 6 software.

### Statistical analysis

Statistical analysis was performed using the SPSS statistical package (version 18.0). The results of quantitative data were expressed as the mean ± SD and evaluated using the Student’s t-test. For Notch1 protein localization quantification, 100 cells from each cell line were counted and the values were calculated from three independent experiments. Pearson’s Chi square test was used to verify the difference. All experiments were performed at least three times. All analyses were two-sided, and *p* < 0.05 was considered statistically significant (*p < 0.05, **p < 0.01, and ***p < 0.001).

## Results

### Notch1^C1133Y^ mutation inactivates the Notch1 signaling pathway

Although missense mutations in Notch1 have previously been identified in OSCC patients in various populations [8, 10], no functional analysis of any Notch1 alleles associated with OSCC has been elucidated.

**Fig. 1:**
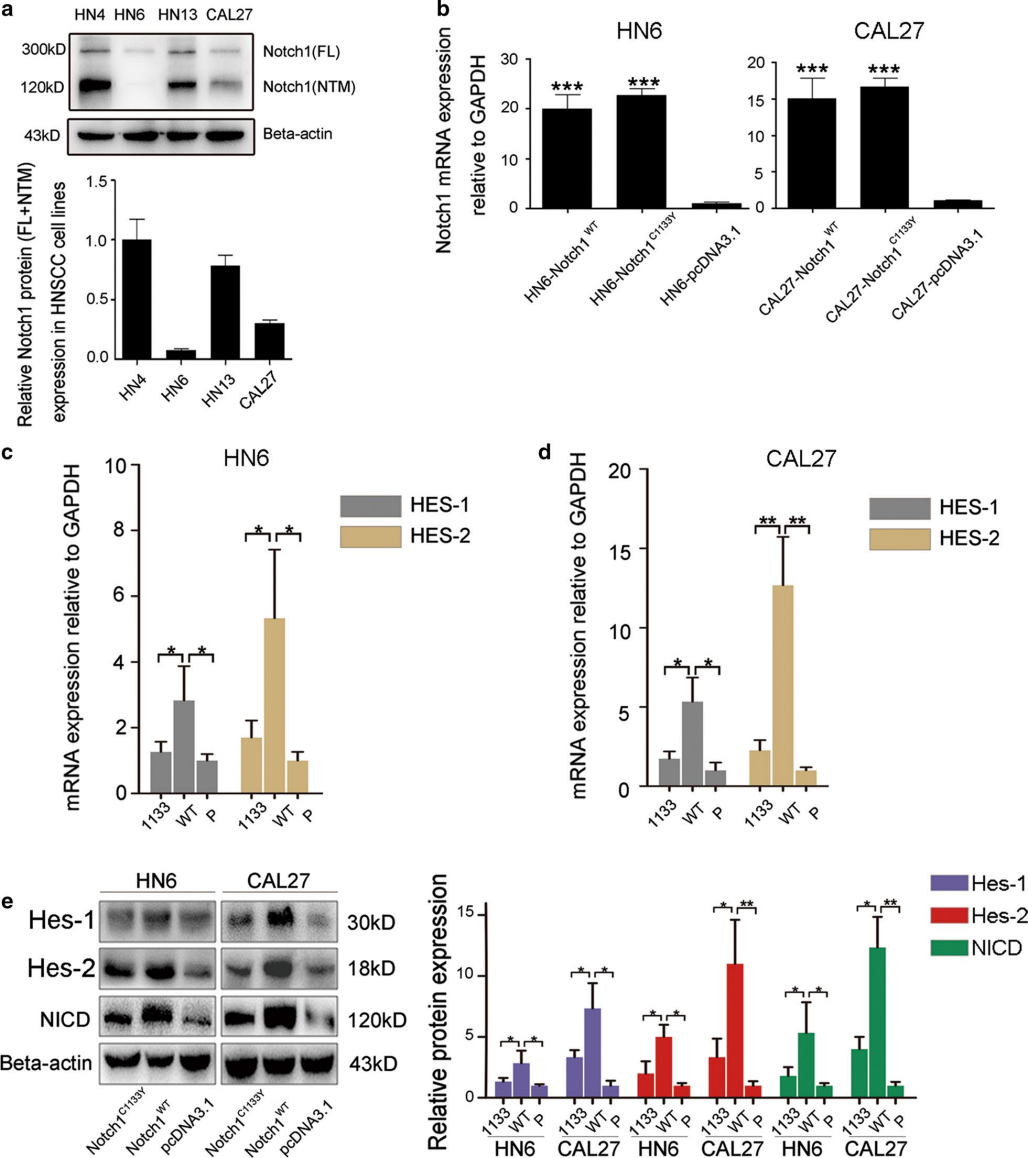
Notch1^C1133Y^ mutation inactivates the Notch1 signaling pathway. **a** Endogenous Notch1 expression in HNSCC cell lines (HN4, HN6, HN13 and CAL27) assessed by western blot analysis (up) and the quantification of the protein expression (down). **b** HN6 and CAL27 cells were transfected with pcDNA3.1-Notch1^WT^, pcDNA3.1-Notch1^C1133Y^, or pcDNA3.1 empty vector. 48 h later, Notch1 mRNA expression was detected by real-time qPCR. **c** In HN6 cells, Notch1 target genes HES1-2 mRNA expressions were determined by real-time qPCR after transfection. **d** Notch1 target genes were also tested in CAL27 cells after transfection. **e** Protein expression levels of HES1-2 and cleaved-NICD (or NICD) were determined by western blot after transfection

Here, we selected the C1133Y hotspot mutation and assessed its association with Notch1 function in OSCC cell lines with varying levels of endogenous Notch1 expression. Western blot analysis revealed that the level of endogenous Notch1 differed across cell lines. HN4 and HN13 cells demonstrated relatively higher Notch1 expression, while CAL27 cells showed moderate expression. Nearly undetectable Notch1 expression was observed in HN6 cells (Fig. 1a). To establish specific Notch1 expression cell lines, HN6 and CAL27 cells were transfected with pcDNA3.1-Notch1^WT^, pcDNA3.1-Notch1^C1133Y^, or pcDNA3.1 empty vector. Forty-eight hours later, transiently transfected HN6-/CAL27-Notch1^C1133Y/WT^ or -pcDNA3.1 cell lines were obtained and verified by real-time qPCR using common primers targeting Notch1 mRNA not covering the mutant nucleotides (Fig. 1b).

We first examined Notch1 signaling pathway activation. Because HES family genes have been reported to be the regular target genes activated by Notch1 signaling [13], we performed real-time qPCR to selectively test the status of HES1-2 genes in HN6 and CAL27 cells after transfection. In HN6 cells, wild-type Notch1 induced downstream target genes up-regulated, compared to the control cells. When compared to the cells transfected with wild-type Notch1, cells transfected with the Notch1 C1133Y mutation induced HES1-2 mRNA down-regulated (Fig. 1c). This trend was similar in Notch1^C1133Y^-transfected CAL27 cells with HES1-2 mRNA being down-regulated, though wild-type Notch1 in CAL27 cells did not activate distinct downstream target genes (Fig. 1d). Reduced HES-1 and HES-2 protein levels were also verified by western blot analysis (Fig. 1e). To verify the Notch1 signaling status, cleaved-NICD, which is the key component of Notch1 signaling and serves as a marker of ‘canonical Notch1 pathway’ activation [6], was also evaluated by western blot analysis. As anticipated, the expression level of cleaved-NICD in Notch1^C1133Y^ cells was much lower than that in Notch1^WT^ or pCDNA3.1 in HN6 and CAL27 cells (Fig. 1e). Taken together, these results strongly suggest that the C1133Y mutation in Notch1 inactivates the Notch1 signaling pathway.

### Notch1^C1133Y^ mutation accelerates cell proliferation

**Fig. 2:**
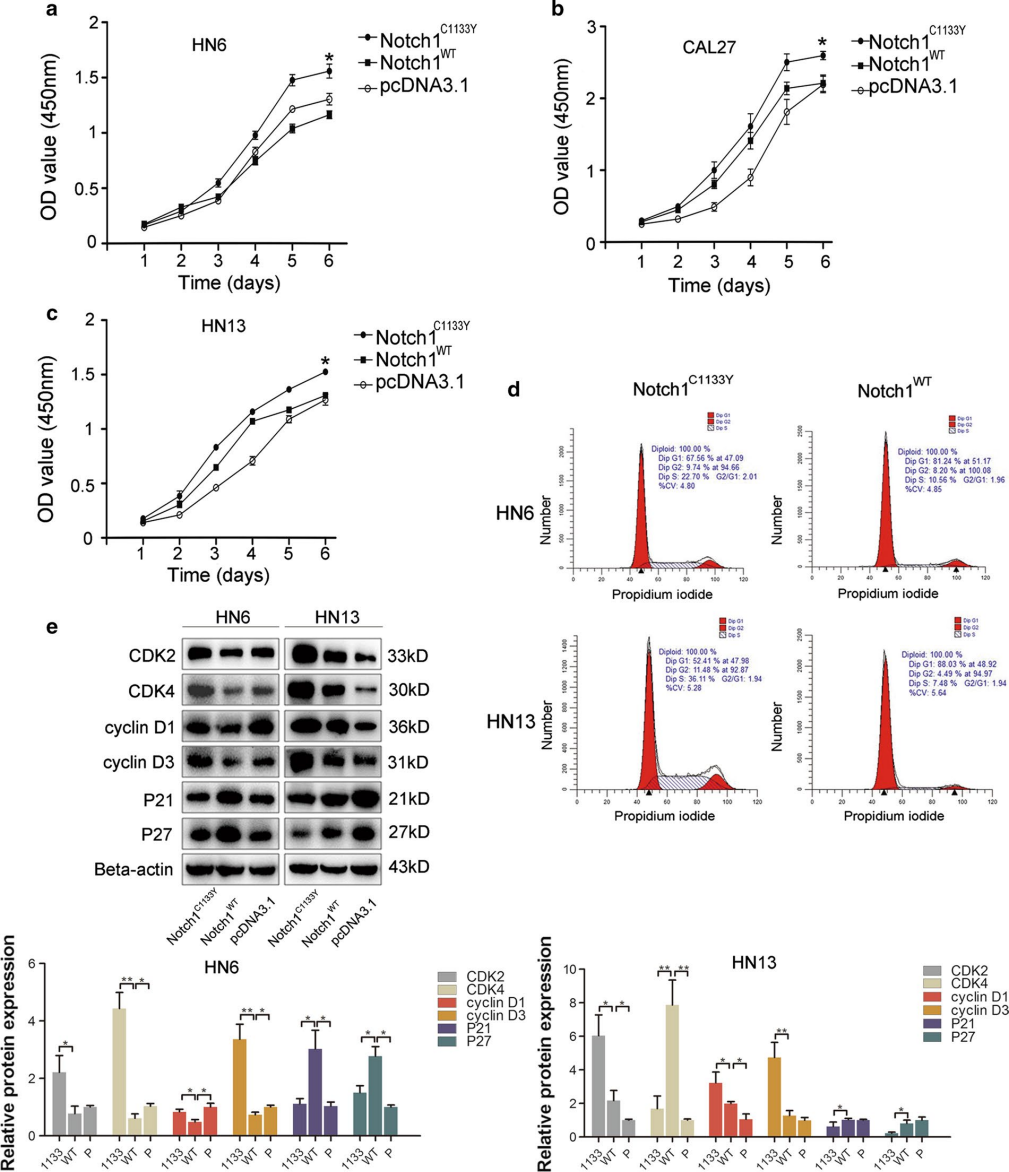
Notch1^C1133Y^ mutation accelerates cell proliferation. **a**–**c** The CCK-8 growth curves of HN6, CAL27 and HN13 cell lines after transfections. The overexpression of wild-type Notch1 attenuated cell growth in HN6, but it enhanced cell growth in CAL27 and HN13, compared with controls. However, Notch1 C1133Y mutation accelerated cell growth in all the tested cell lines, compared with Notch1^WT^-transfected cells. **d** The cell-cycle analysis by flow cytometry revealed a less G1 phase arrest in Notch1^C1133Y^-transfected cells. **e** Cell-cycle-specific proteins were analyzed by western blot analysis in HN6 and HN13. Notch1^C1133Y^ transfection resulted in the up-regulation of CDKs (2 and 4) and cyclins (D1 and D3), whereas the expression of P27 and P21 was decreased, suggesting an expedited cell-cycle induced by Notch1^C1133Y^ transfection. EGFR-PI3K/AKT signaling activities were evaluated by western blot analysis. Except that EGFR and AKT levels remained unchanged, wild-type Notch1 decreased the expression levels of p-EGFR, p-Stat5, p-Shc, and p-Gab1, while the Notch1^C1133Y^ mutation reversed the trend in all the tested cell lines. Notch1^C1133Y^ mutation increased p-AKT and PI3K levels in all the tested cell lines, though the p-AKT and PI3K expressions were down-regulated in HN6 or up-regulated in CAL27 and HN13 induced by wild-type Notch1 transfection

To test the effect of the Notch1 C1133Y mutation on cell proliferation, CCK-8 assays were utilized. As shown in Fig. 2a–c, the CCK-8 growth curves indicated that the Notch1^C1133Y^-transfected cells proliferated more frequently compared with the Notch1^WT^-transfected cells. Moreover, we performed cell-cycle analysis by flow cytometry. The results demonstrated that Notch1^WT^-transfected cells presented a significantly higher percentage of cells in the G1 phases (81% in HN6 cells or 88% in HN13 cells) than the Notch1^C1133Y^-transfected cells (68% in HN6 cells or 52% in HN13 cells). Meanwhile, Notch1^WT^-transfected cells showed a lower percentage of cells in the S phase (10% in HN6 cells or 7% in HN13 cells) than the Notch1^C1133Y^-transfected cells (23% in HN6 cells or 36% in HN13 cells) (Fig. 2d). These results demonstrated a less G1 phase arrest induced by Notch1^C1133Y^ transfection, compared to Notch1^WT^-transfected cells. In addition, changes in the expression of cell-cycle-specific proteins were also analyzed by western blot analysis [29]. As expected, Notch1^C1133Y^ transfection resulted in the up-regulation of cyclin-dependent kinases (CDKs) (2 and 4) and cyclins (D1 and D3), whereas the expression of P21 and P27 was decreased (Fig. 2e). All these results suggest that the Notch1^C1133Y^ mutation expedites the proliferative ability by accelerating the cell-cycle.

Cell apoptosis using cleaved Caspase-3 and Flow cytometry was also tested in this study (Additional file 1: Figure S1A–B). In HN6 and HN13 transfected cells, no apparent discrepancy has been discovered between wild-type Notch1 and C1133Y-mutant Notch1.

### Notch1^C1133Y^ mutation enhances cell invasion and regulates EMT markers

**Fig. 3:**
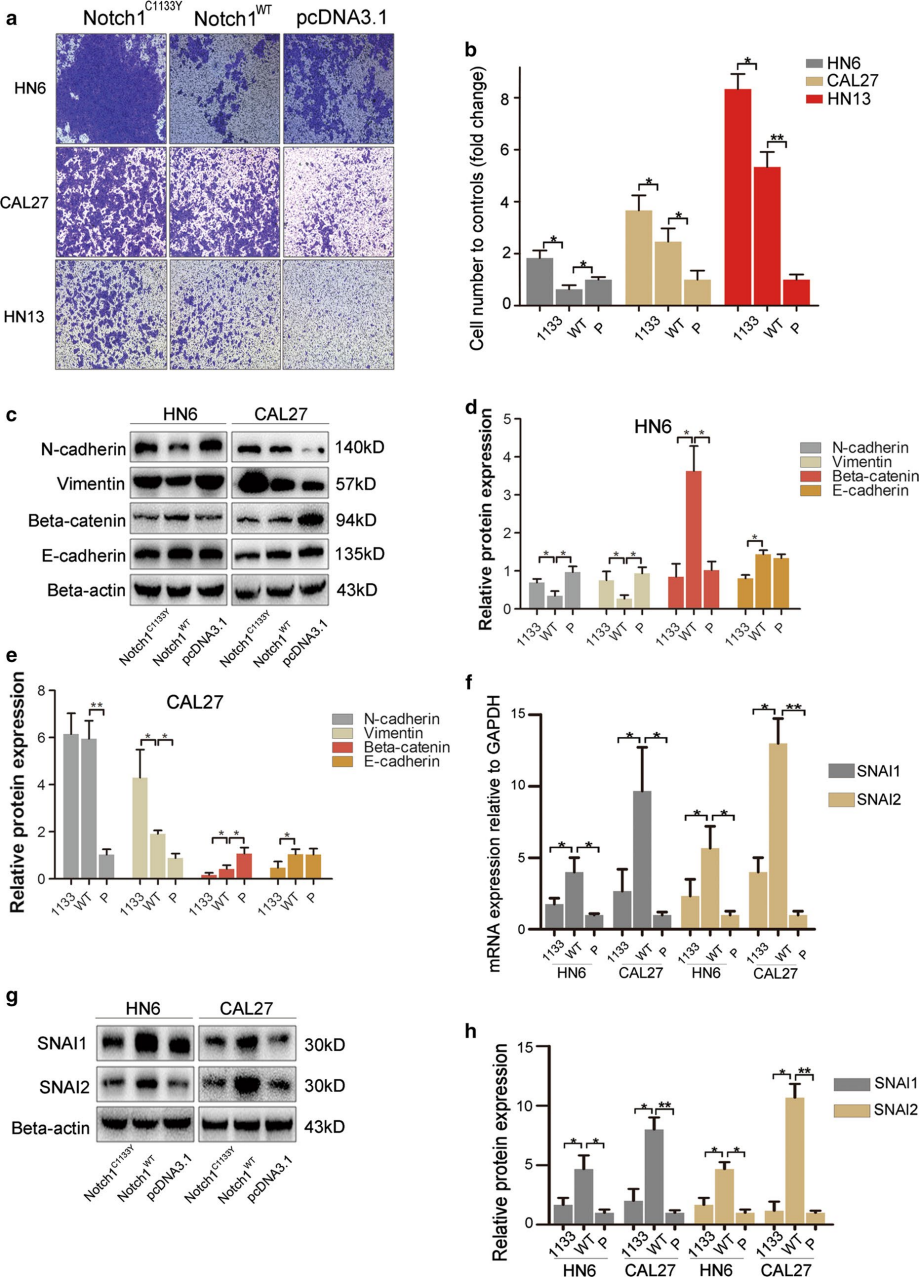
Notch1^C1133Y^ mutation enhances cell invasion and regulates EMT markers. **a** Images of Transwell invasion assays (left) and the quantification of the cells (right). Each data point represents the mean ± SD of data from 3 independent trials. **b** Quantitive analysis of **a**. **c** Western blot analysis was used to assess the expression of epithelial (E-cadherin and β-catenin) and mesenchymal (N-cadherin and Vimentin) markers in Notch1^C1133Y^- or Notch1^WT^-transfected cell lines (HN6 and CAL27). **d**, **e** The greyscale analysis of panel C. **f** Real-time qPCR was used to detect the expression of classic EMT-inducer Snail (SNAI1, SNAI2) mRNA in Notch1^C1133Y^- or Notch1^WT^-transfected cell lines (HN6 and CAL27). **g** Western blot analysis was performed to assess the expression of Snail (SNAI1, SNAI2) protein after transfections. **h** The greyscale analysis of panel G

Notch has been reported to promote epithelial-to-mesenchymal transition (EMT) during tumor progression [30]. During the process of EMT, cells acquire more mesenchymal cell properties, exhibiting higher invasive ability. To determine if Notch1^C1133Y^ mutation also effects invasiveness in OSCC cells, we performed Transwell invasion assays. As shown in Fig. 3a, b, Notch1^WT^-transfected cells demonstrated higher invasive ability in CAL27 and HN13 cells but lower invasive ability in HN6 cells compared with controls. In addition, our results revealed that Notch1^C1133Y^-transfected cells exhibited even higher invasive ability in all three cell lines compared with Notch1^WT^-transfected cells.

Notch1 promotes EMT during cardiac development, involving down-regulation of epithelial markers (E-cadherin) and up-regulation of mesenchymal markers (Snail, Vimentin, and c-Myc) [31–33]. Notch1 mutations acting through a similar mechanism may also be involved in the EMT process of OSCC cells. Therefore, we utilized western blot analysis to assess the protein expression levels of EMT-specific markers. As expected, transfection of Notch1^WT^ induced up-regulation of mesenchymal markers (N-cadherin and Vimentin) in CAL27 cells and down-regulation of these markers in HN6 cells, whereas the expression of the epithelial marker Beta-catenin was decreased in CAL27 cells or increased in HN6 cells (Fig. 3c−e) compared with controls. There was no difference in E-cadherin expression between the two groups in both cell lines. An increased expression of mesenchymal markers (N-cadherin and Vimentin) and a decreased expression of the epithelial marker Beta-catenin were observed in Notch1^C1133Y^-transfected CAL27 and HN6 cells compared with Notch1^WT^-transfected cells, thus verifying its enhanced status of EMT induced by the C1133Y mutation.

Notch1 can also enhance EMT during tumor progression via crosstalk with several regulators and growth factors relevant to EMT [20, 33, 34]. The Snail, Zeb, and cMET transcription factors are representative EMT regulators, and they have been previously implicated as inducers of EMT in many types of malignancies [17, 34]. To further elucidate the potential molecular mechanisms of EMT induced by the C1133Y mutation, we analyzed the gene expression levels of the classic EMT-inducers, Zeb (Zeb1 and Zeb2) and Snail (SNAI1 and SNAI2), by real-time qPCR. As shown in Fig. 3f, in both HN6 and CAL27 cells, an increase in SNAI1/2 gene expression was observed in Notch1^WT^-transfected cells, while the expression of Zeb1 and Zeb2 was not significantly different in Notch1^WT^-transfected cells compared with controls (data not shown). Astonishingly, decreased expression of SNAI1/2 was observed in Notch1^C1133Y^-transfected cells, which was not consistent with the EMT phenotype. Altered SNAI1 and SNAI2 protein expression was also verified by western blot analysis (Fig. 3g, h). Taken together, these data suggested that Notch1^C1133Y^ mutation induces cell invasion and EMT in HNSCC cell lines but down-regulates SNAI1/2 expression.

### Notch1^C1133Y^ mutation causes reduced S1-cleavage of the Notch1 receptor and accumulation of receptor protein in the endoplasmic reticulum

**Fig. 4:**
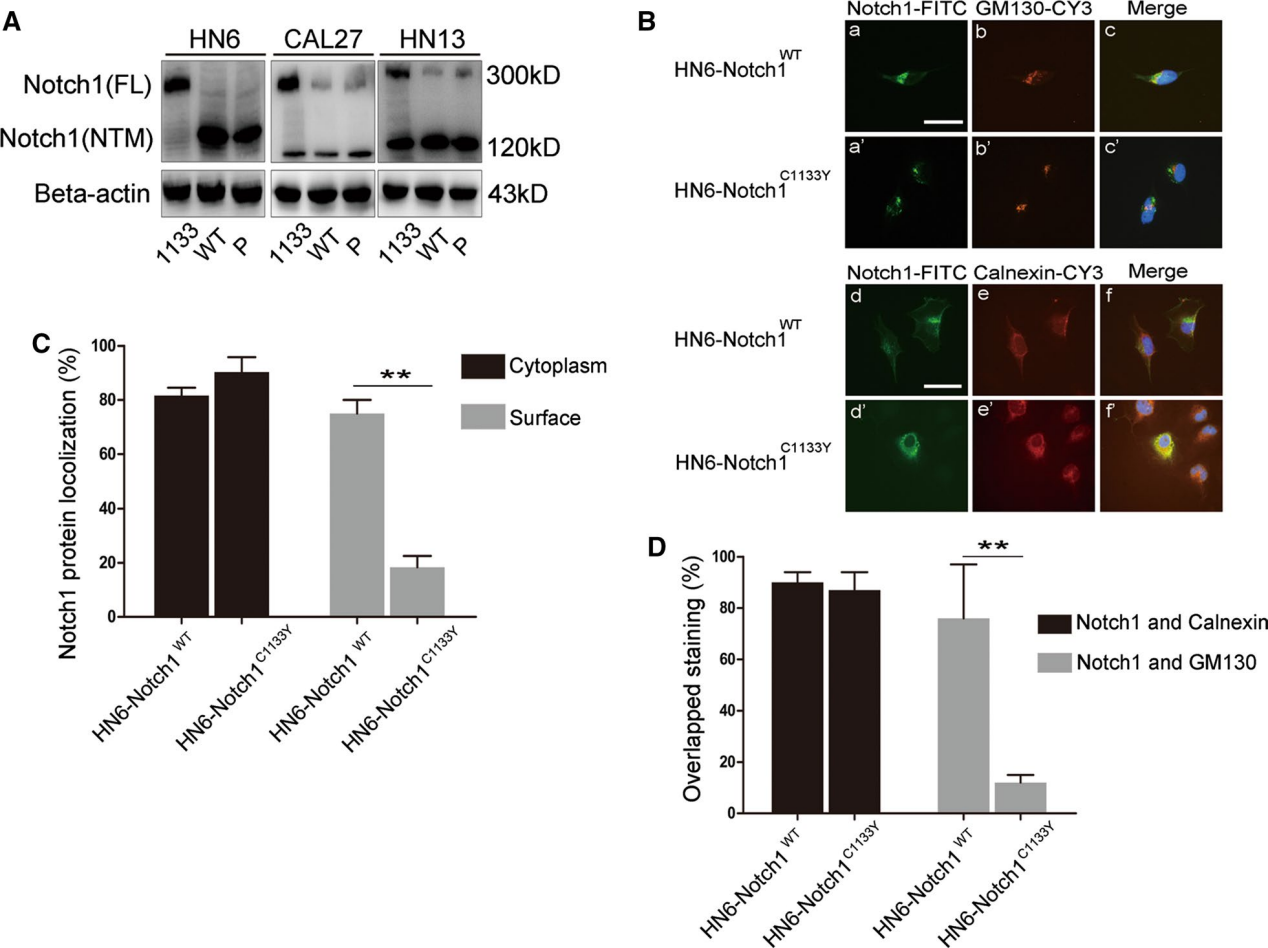
Notch1^C1133Y^ mutation causes reduced S1-cleavage of the Notch1 receptor and accumulation of receptor protein in the endoplasmic reticulum. **A** Notch1 protein expression pattern was evaluated by western blot using a Notch1 primary antibody which could detect endogenous Notch1 protein and recognize both full-length Notch1 (FL, 300 kD, S1-uncleaved premature form) and its cleaved transmembrane/intracellular region (NTM, 120 kD, S1-cleaved and S2/S3-cleaved form). **B** The subcellular location of Notch1 receptors in HN6 cells was assessed by immunofluorescence. The Notch1-FITC staining revealed that Notch1 protein in Notch1^WT^-transfected cells was localized in the cytoplasm as well as on the cell surface (**a**, **d**), while Notch1 protein in C1133Y-mutated cells was only localized in the cytoplasm (**a′**, **d′**). Costaining of Notch1 with the Golgi-marker GM130 (**b**, **b′**) demonstrated overlapped staining in Notch1^WT^-transfected cells (**c**), but did not showed overlap in Notch1^C1133Y^-transfected cells (**c′**). Costaining of Notch1 with ER-marker Calnexin (**e**, **e′**) showed strong overlapped staining in both Notch1^WT^ and Notch1^C1133Y^-transfected cells (**f**, **f′**). Scale bars are 10 μm. **C** The localization of Notch1 in cytoplasm or on cell surface was assessed in 100 cells, and the percent of cells was shown. **D** Overlapped staining of Notch1 with Golgi-marker GM130, or Notch1 with ER-marker Calnexin was counted. Percentages of localization were calculated from three independent experiments

The data in Fig. 1 demonstrated a decreased cleaved-NICD expression after transfection of Notch1^C1133Y^ compared to Notch1^WT^. One possible explanation for this could be that the C1133Y mutation induces functionally inert S3-cleavage, releasing less Notch1 intracellular domain. Other explanations lie in all the conditions that produce less structurally or functionally mature Notch1 protein. As S1-cleavage in Golgi complex has been described as required for canonical ligand-dependent Notch1 signaling prior to its presentation to the cell surface, the 120 kD band represents Notch1 protein that has undergone S1- or S2-/S3-cleavage, while the 300 kD band is expected to be the full-length Notch1 protein that has not undergone S1-cleavage [35]. We performed western blot analysis to evaluate the Notch1 protein expression pattern using the Notch1 primary antibody that detects endogenous Notch1 protein and recognize both full-length (300 kD) Notch1 and its cleaved intracellular region (120 kD). In HN6 cells, which express low levels of endogenous Notch1, Notch1 protein was detected mostly in the 300 kD form after transfection with Notch1^C1133Y^. In Notch1^WT^-transfected HN6 cells, nearly all the Notch1 protein was in the 120 kD form. For cells (CAL27 and HN13) expressing relatively moderate or high levels of Notch1, both the 120 and 300 kD forms of Notch1 protein were detected after transfection with Notch1^C1133Y^, while the 120 kD form and a small amount of the 300 kD form were presented in Notch1^WT^ and pcDNA cells (Fig. 4a). These data strongly imply that the C1133Y mutation causes reduced S1-cleavage.

Only S1-cleaved Notch1 receptors can be sent to the cell surface [36]. Thus the observed reduced S1-cleavage caused by the Notch1 C1133Y mutation may be attributed to either retention of Notch1 protein in the endoplasmic reticulum (ER) and failure of transporting to the Golgi complex or the functional impairment of S1-cleavage in the Golgi complex. To test this hypothesis, we performed immunofluorescence (Notch1-FITC) to visualize the intracellular localization of Notch1 protein [37, 38]. In HN6 cells, Notch1 protein in Notch1^WT^-transfected cells was localized in the cytoplasm and on the cell surface (Fig. 4B a, d). In contrast, Notch1 protein in C1133Y-mutated cells was mostly detected in the cytoplasm (Fig. 4B a′, d′). Quantification of Notch1 expression localization showed an obvious difference between the two groups (Fig. 4C). The data indicated that 80% of Notch1^WT^-transfected cells and 87% of Notch1^C1133Y^-transfected cells exhibited cytoplasmic expression of Notch1, but that only 17% of Notch1^C1133Y^-transfected cells exhibited cell surface expression compared with 76% of Notch1^WT^-transfected cells exhibiting cell surface expression. Furthermore, we used a Golgi-specific marker (GM130-CY3) and an endoplasmic reticulum-specific marker (Calnexin-CY3) with immunofluorescence to define Notch1 protein localization. Consistent with the Notch1-FITC results, 90% of Notch1^WT^-transfected cells (Fig. 4B d, e, f) and 86% of Notch1^C1133Y^-transfected cells (Fig. 4B d′, e′, f′) showed overlap between Notch1-FITC and the ER marker, while 76% of Notch1^WT^-transfected cells (Fig. 4B a, b, c) and only 12% of Notch1^C1133Y^-transfected cells (Fig. 4B a′, b′, c′) showed overlapped staining between Notch1-FITC and the Golgi marker (*p* < 0.01, Fig. 4D). These findings strongly suggest that transport of Notch1 protein in Notch1^C1133Y^-transfected cells from the ER to Golgi is impaired, leading to less protein transportation to the Golgi for S1-cleavage and ultimately less protein on the cell surface.

### Notch1^C1133Y^ mutation activates the EGFR-PI3K/AKT signaling pathway

**Fig. 5:**
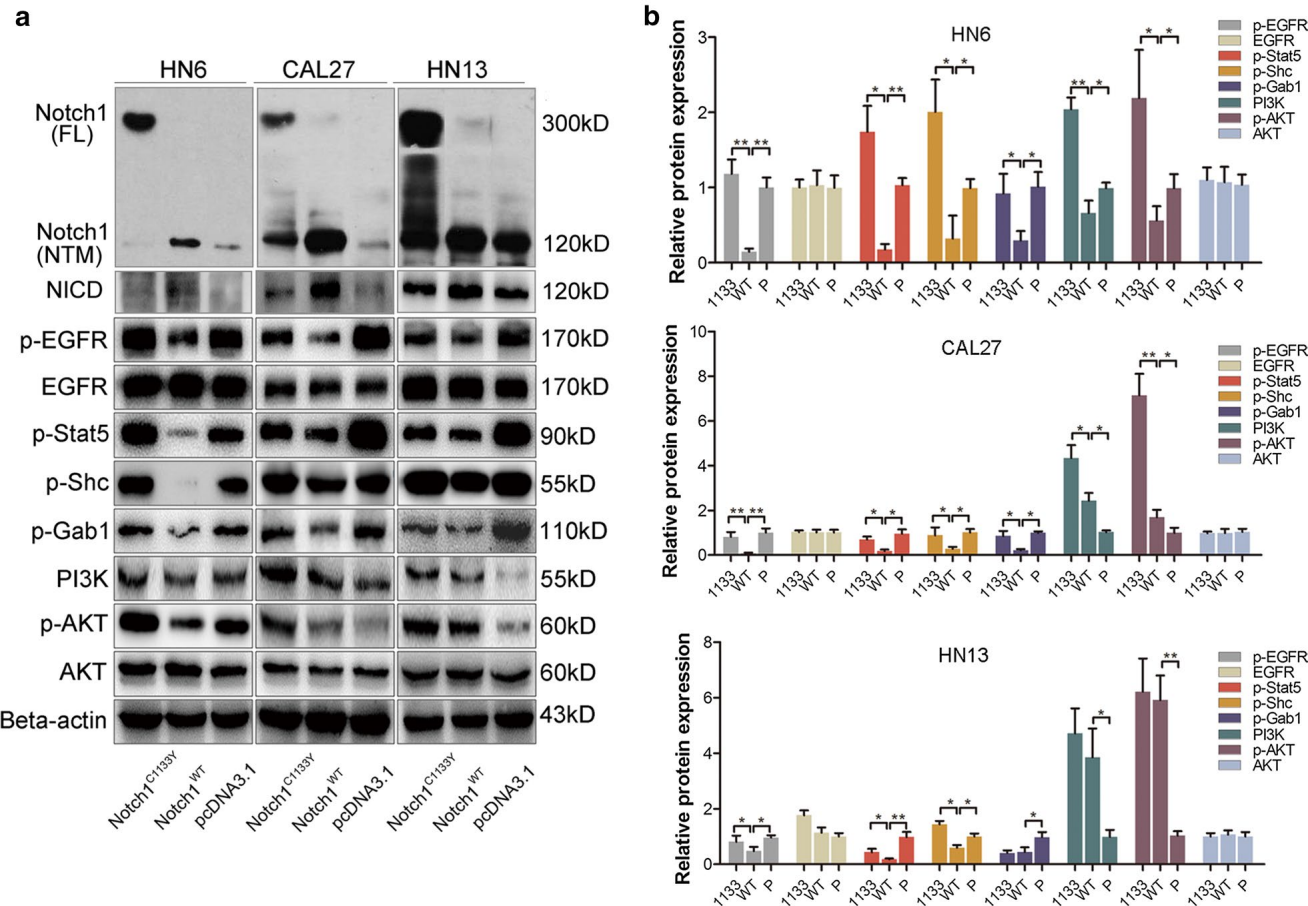
Notch1 C1133Y mutation activates the EGFR-PI3K/AKT signaling pathway. **a** The efficiency of Notch1 transfection was verified in HN6, CAL27 and HN13 cells. EGFR-PI3K/AKT signaling activities were evaluated by western blot analysis. Except that EGFR and AKT levels remained unchanged, wild-type Notch1 decreased the expression levels of p-EGFR, p-Stat5, p-Shc, and p-Gab1, while the Notch1^C1133Y^ mutation reversed the trend in all the tested cell lines. Notch1^C1133Y^ mutation increased p-AKT and PI3K levels in all the tested cell lines, though the p-AKT and PI3K expressions were down-regulated in HN6 or up-regulated in CAL27 and HN13 induced by wild-type Notch1 transfection. **b** The grey-scale analysis of the alteration of EGFR pathway in panel A

Overexpression of EGFR, which has been reported in up to 30% of solid tumors including 90% of OSCC [39, 40], generally correlates with a poor prognosis and promotes tumor proliferation. Notch1 has also been revealed to have an interplay with EGFR [26, 41, 42]. Thus, we investigated EGFR signaling activities using EGF receptor pathway-specific antibodies (including EGFR, p-EGFR, AKT, p-AKT, PI3K, p-Stat5, p-Shc, and p-Gab1) by western blot analysis. In all three cell lines, EGFR and AKT levels remained unchanged. The expression levels of p-EGFR, p-Stat5, p-Shc, and p-Gab1 decreased after cells were transfected with Notch1^WT^. An opposite pattern was observed after the Notch1^C1133Y^ mutation was introduced (Fig. 5a). Moreover, Notch1^WT^ transfection induced down-regulated p-AKT and PI3K in HN6 cells or up-regulated p-AKT and PI3K in CAL27 and HN13 cells. p-AKT and PI3K were increased in all three cell lines after cells were transfected with Notch1^C1133Y^, compared with the Notch1^WT^ transfection (Fig. 5a). Figure 5b showed the grey-scale analysis of the alteration of EGFR pathway in panel A.

## Discussion

**Fig. 6:**
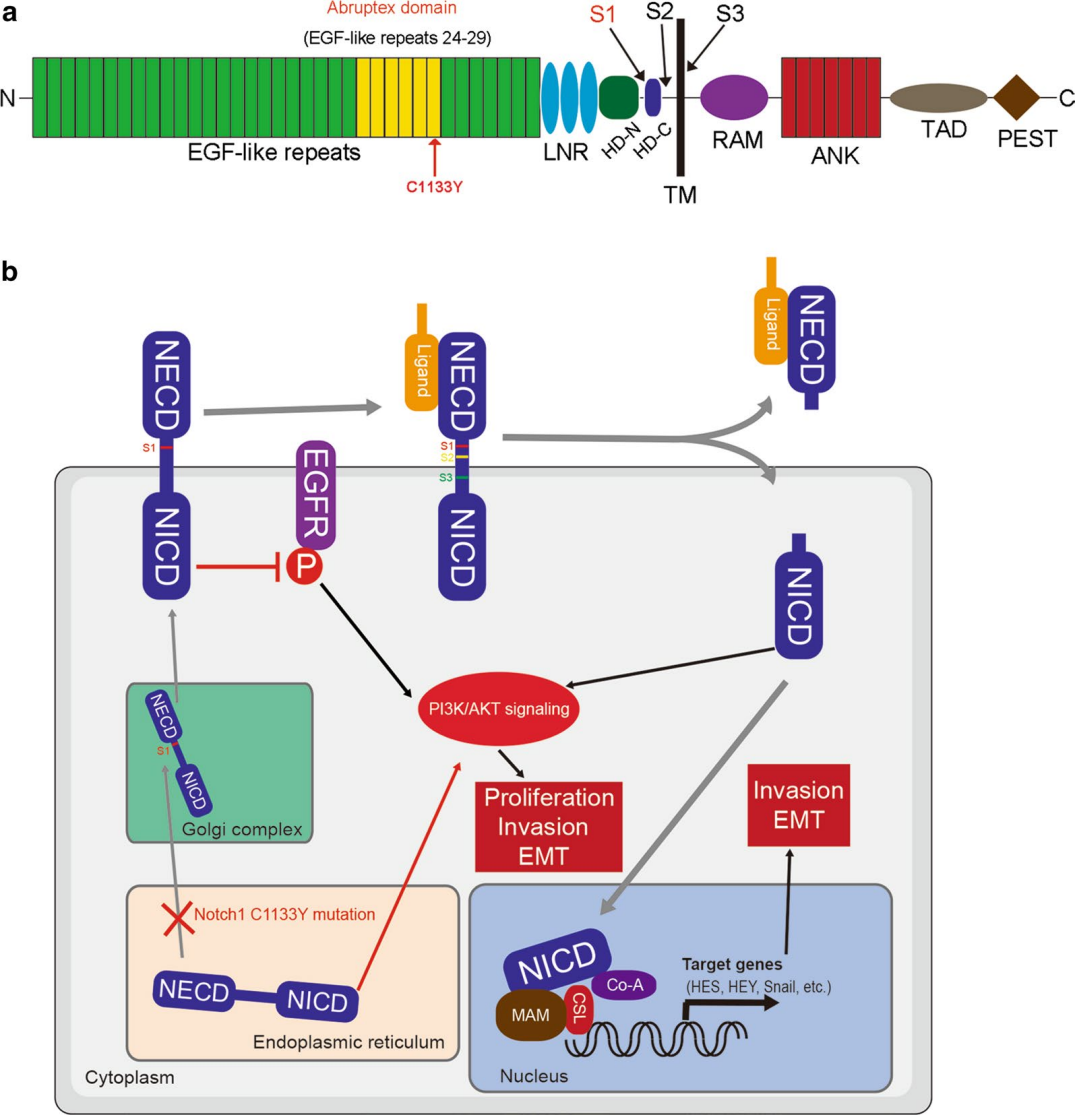
Schematic model for EGFR-PI3K/AKT signaling activation induced by Notch1^C1133Y^ mutation. **a** Schematic depiction of Abruptex domain C1133Y mutation and S1-3 cleavages in Notch1 receptor protein. *EGF* epidermal growth factor, *LNR* Lin/Notch repeats, *HD* (N and C regions), heterodimerization domain, *TM* transmembrane domain, *RAM* RBP-Jκ-associated molecule region, *ANK* ankyrin repeats, *TAD* transactivation domain, *PEST* sequence rich in proline, glutamic acid, serine, and threonine. S1-3, S1-3 cleavages. Black arrows indicate the sites of the cleavages. Red arrow indicates the site of the C1133Y mutation. **b** Model for aberrant EGFR-PI3K/AKT signaling pathway activation by Notch1 C1133Y mutation. The Notch1 protein is synthesized in endoplasmic reticulum and is transported to Golgi complex for S1-cleavage. The S1-cleaved mature Notch1 protein is presented on cell surface, where it has an inhibitory effect on EGFR phosphorylation. The ligand binding causes cleavage of the receptor at the S2-cleavage site. The remaining Notch1 receptor undergoes further cleavage at the S3 site, freeing the NICD domain. The NICD translocates to the nucleus where it binds to the DNA-binding protein CSL and was recognized by the transcriptional coactivator Mastermind (MAM). The triprotein complex recruits additional coactivators (Co-A) to activate target genes. In this study, we find that the Notch1 signaling has an inhibitory effect on EGFR activation. When Notch1 C1133Y mutation occurs, Notch1 protein is arrested in endoplasmic reticulum and is unable to be transported to Golgi complex for S1-cleavage, thus the canonical Notch1 signaling activation is disrupted. The PI3K/AKT signaling is activated by Notch1 protein arrest in endoplasmic reticulum induced by Notch1 C1133Y mutation. Moreover, the loss of inhibitory effect by Notch1 loss-of-function mutation can also induces EGFR phosphorylation, thus activating PI3K/AKT signaling. *NECD* Notch1 extracellular domain, *NICD* Notch1 intracellular domain

Recently, deep genome sequencing of cancers has produced a substantial amount of data involving Notch signaling in human malignancies, including OSCC. Since then, a substantial number of functional mutations have been discovered. In our previous study, we sequenced the entire coding region of the Notch1 gene in 51 OSCC tissues from a Chinese population and discovered that 13 (31%) mutations (including three nonsense mutations and a C1133Y hotspot mutation in three tumors) in the Abruptex region (Fig. 6a). The mammalian Notch extracellular domain has 36 EGF repeats, and six of which (repeats 24–29) are detected by a series of Notch missense mutations in Drosophila, the called Abruptex domain [43]. These mutations result in amino-acid substitutions in the Abruptex domain and induce phenotypes opposite to those characteristics of decreased Notch expression during Drosophila development [44, 45]. For example, in sensory organ formation and vein differentiation, the Abruptex domain alleles are associated with the loss of these structures, whereas Notch1 loss-of-functional alleles cause the formation of extra sensory organs and vein tissue. Jose et al. [28] has determined that Abruptex alleles identify a domain in the Notch protein that mediates the interactions among Notch, its ligands and Fringe that result in suppression of Notch activity, suggesting that the Abruptex domain mediates interactions between Notch and other proteins that inhibit Notch signaling activity. Zifei P et al. [27] utilize protein-binding assays and determines that in addition to binding to the ligand-binding region (EGF repeats 11–12), Delta can also bind to EGF repeats 22–27 of Notch, which overlap the Abruptex domain. As a result, the Abruptex domain competes with Delta for binding to the ligand-binding domain. Thus, the Abruptex domain may acts as a negative regulator of Notch signaling activation, and any missense mutation in this region may reverse the antagonistic effects on Notch signaling activation.

To verify the activation of Notch1 pathway, we first tested downstream signaling using western blot and real time-qPCR in cells transfected with pcDNA3.1-Notch1^WT^, pcDNA3.1-Notch1^C1133Y^, or pcDNA3.1 empty vector. Results showed Notch1C1133Y mutation inactivated Notch1 pathway. Further, CCK-8 and Transwell assays were performed in the experiment. Compared with cells transfected with Notch1^WT^, cells with Notch1^C1133Y^ showed enhanced proliferative and invasive ability.

To detect the molecular mechanisms that may underlie the loss-of-function in Notch1 signaling through the C1133Y mutation, we examined Notch1 protein expression and localization. Notch1^C1133Y^-mutant cells exhibited both reduced S1-cleavage and cell surface receptor level. Our findings further revealed that S1-uncleaved immature Notch1 protein localized to the ER in majority of Notch1^C1133Y^-mutant cells, which contrasted with the usual Notch1 protein localization in Golgi complex and on the cell surface. These data may explain why the estimated gain-of-function mutation in Abruptex domain observed in transient cells adversely inactivated the Notch1 signaling in stable cells: the unexpected inactivation of Notch1 ligand-induced signaling was due to the retention or misfolding of Notch1 protein in the ER, which would lead to reduced transportation of full-length Notch1 protein from the ER to the Golgi complex for presumed S1-cleavage and ultimately presence on the cell surface, on which way the Notch1 signaling pathway was inactivated. Previous evidence has suggested that missense mutations in EGF repeats, not in the Abruptex domain, can cause Notch1 protein retention or misfolding. For example, a similar study [37] has found that a Notch1^A683T^ mutation (in EGF repeats 18) in left ventricular outflow tract defect patients causes deficient Notch1 protein localization induced by receptor retention in the ER. All these evidences hint that Notch1^C1133Y^ mutation leads to an ‘Abruptex-specific’ loss-of-function of Notch1 signaling.

Until now, there has been no functional analysis of Abruptex domain mutations in pathological diseases, such as carcinoma. In this study, we selected the C1133Y Abruptex domain mutation and examined its functional effects on Notch1 signaling in OSCC. We found that Notch1^C1133Y^ transiently activated the Notch1 signaling pathway in OSCC cell lines compared with Notch1^WT^-transfected cells, after which Notch1^C1133Y^ induced Notch1 signaling inactivated in stable cell lines. These data gave rise to evidence that C1133Y in Abruptex domain could be an ‘Abruptex-specific’ mutation (as mentioned above) as expected.

We detected the oncogenic phenotype alterations and found that over-expression of wild-type Notch1 had diverse effects on cell proliferation. Notch1 enhanced cell proliferation in CAL27 and HN13 cells, and it compromised cell proliferation in HN6 cells. We speculate that the interplay between the Notch1 and EGFR signaling pathways as well as the endogenous Notch1 level were responsible for these differences. Several studies have focused on the complicated cross-talk among Notch1, EGFR and their common downstream PI3K/AKT signaling, and they have revealed that the repressive interplay between EGFR and Notch1 could act as compensatory effectors towards the downstream targets, such as the AKT/PI3K pathway [26, 41, 42]. Specifically, communication between the Notch and EGFR pathways in cancer cells enables the cells to compensate for the loss of one pathway with the increase in the other. In HN6 cells, which had negligible endogenous Notch1 expression and a substantial amount of EGFR expression (data not shown), transfection of wild-type Notch1 did not overtly affect Notch1 pathway activation as distinct Notch1 over-expression did not evidently alter its downstream target genes. In HN13 cells, Notch1 signaling was drastically activated by wild-type Notch1 transfection, predicting the Notch1-dominant status in HN13, as well as the EGFR-dominant status in HN6. Because PI3K/AKT signaling is primarily responsible for cell proliferation and invasion in malignancies [46–48], in the EGFR-dominant HN6 cell line, inhibition of EGFR-AKT induced by Notch1^WT^ transfection induced an attenuated proliferative and invasive capacity, while in the Notch1-dominant HN13 cells, the activated Notch1 pathway mainly contributed to the enhanced cell invasion. The activated AKT pathway induced by Notch1^WT^ transfection also played a role in cell proliferation and invasion. Another contradiction in this study lies in the fact that in CAL27 or HN13 cells, Notch1^WT^ led to enhanced cell proliferation and invasion, while Notch1^C1133Y^ loss-of-function mutation still induced enhanced proliferative and invasive ability. We speculated that this inconsistence was mainly attributed to the role of the AKT pathway. Notch1-mediated activation of AKT signaling has been poorly reported. Several studies have indicated that AKT signaling could be activated by the activated form of Notch1 (NICD) [49–51] and is CSL-independent [52]. In this work, however, we found that AKT signaling could be activated by the 300 kD form of retarded Notch1 protein induced by Notch1^C1133Y^ mutation. These data indicate that AKT could be activated by Notch1 post-translationally and does not require ligand-dependent membrane cleavage of Notch1. Nevertheless, it remains unknown how 300 kD form of Notch1 in ER affects cytoplasmic signaling of PI3K-AKT. It is doubtful if the C1133Y mutation-induced AKT activation is attributed to Notch1 retention in cytoplasm directly or to the loss of inhibitory effect on EGFR phosphorylation. It’s still unclear whether the opposite effect on oncogenic phenotypes induced by C1133Y loss-of-function mutation is AKT signaling-dependent, thus requiring further investigation.

Another implication of this work is the explicit positive regulation of the Snail family induced by the Notch1 signaling pathway, which is closely related to EMT in HNSCC [53]. In this study, wild-type Notch1 up-regulated Snail expression in both HN6 and CAL27 cells although Notch1^WT^-transfected HN6 cells exhibited a reverse EMT phenotype. Loss-of-function induced by the Notch1^C1133Y^ mutation decreased Snail expression, which was opposite to its gain-of-function manner, but not consistent with the EMT phenotype caused by Notch1^C1133Y^ mutation. The Snail superfamily of zinc-finger transcription factors is reported to be involved in the acquisition of invasive properties during tumour progression [54]. It has also been reported that Notch1 signaling pathway can promote metastasis mediated by Snail/Slug [55] and inhibits the expression of E-cadherin resulting in inhibition of expression of beta-catenin and destabilization of adherens junctions [56]. In Notch1^WT^ transfected CAL27 cells, up-regulation of mesenchymal markers (N-cadherin and Vimentin) and down-regulation of the epithelial marker Beta-catenin was discovered, which was consistent with the Transwell assays. The expression of Snail also coordinated with the alteration of mesenchymal markers, which verified its role in tumor invasion. It can be assumed that Notch1 could induce EMT through Snail activation. However, in Notch1^C1133Y^ transfected cells, although EMT phenotype was demonstrated in all three cell lines, the snail expression was less of which in the Notch1^WT^ cells. Such results implicated that in contrast with Notch1, the Notch1^C1133Y^ mutation-induced EMT was not attributed to the Snail family, thus indicating a complex role for Notch1 mutation in the EMT process. Further experiments would be implemented to uncover the EMT mechanism in this novel Notch1 mutation.

The findings in this work (summarized and illustrated in Fig. 6b) found evidence for the first time that mutation in Notch1 Abruptex domain can have an inactivated effect on Notch1 signaling pathway and promotive effects on cell oncogenic phenotypes. They also consolidate our anticipation that a wide spectrum of OSCCs is genetically relevant and that a single genetic alteration could underlie oncogenic phenotypic outcomes. Our findings support the suggestion that OSCCs share a common genetic cause and depend upon crosstalk with other regulatory pathways.

## Conclusions

Taken together, our data reveal that the Notch1 C1133Y mutation enhances proliferative and invasive ability in OSCC cell lines. This novel Notch1 mutation impairs the processing of notch1 protein, and activates the EGFR-PI3K/AKT signaling.

## Additional file

**Additional file 1: Figure S1.** Cell apoptosis was analyzed in HN6 and HN13 transfected cells. (**A**) Cleaved Caspase-3 was utilized to detect the cell apoptosis in HN6 and HN13 cells. (**B**) Flow cytometry was used to determine the early and late stages apoptotic cells.

## Abbreviations

OSCC: oral squamous cell carcinoma
EMT: epithelial-to-mesenchymal transition
ER: endoplasmic reticulum
NICD: Notch intracellular domain
CBF1: CSL DNA-binding protein
HES: Hairy/Enhancer of Split family
HEY: Hairy/Enhancer-of-Split related with YRPW motif
FBS: fetal bovine serum
BSA: bovine serum albumin
PVDF: polyvinylidene fluoride
EGFR: epithelial growth factor receptor
CDKs: cyclin-dependent kinases
