# Supplementary figures and images for: A novel Notch1 missense mutation (C1133Y) in the Abruptex domain exhibits enhanced proliferation and invasion in oral squamous cell carcinoma
Source: Cancer Cell Int. 2018 Jan 8;18:6. doi: 10.1186/s12935-017-0496-5 (PMC5759178; doi:10.1186/s12935-017-0496-5)

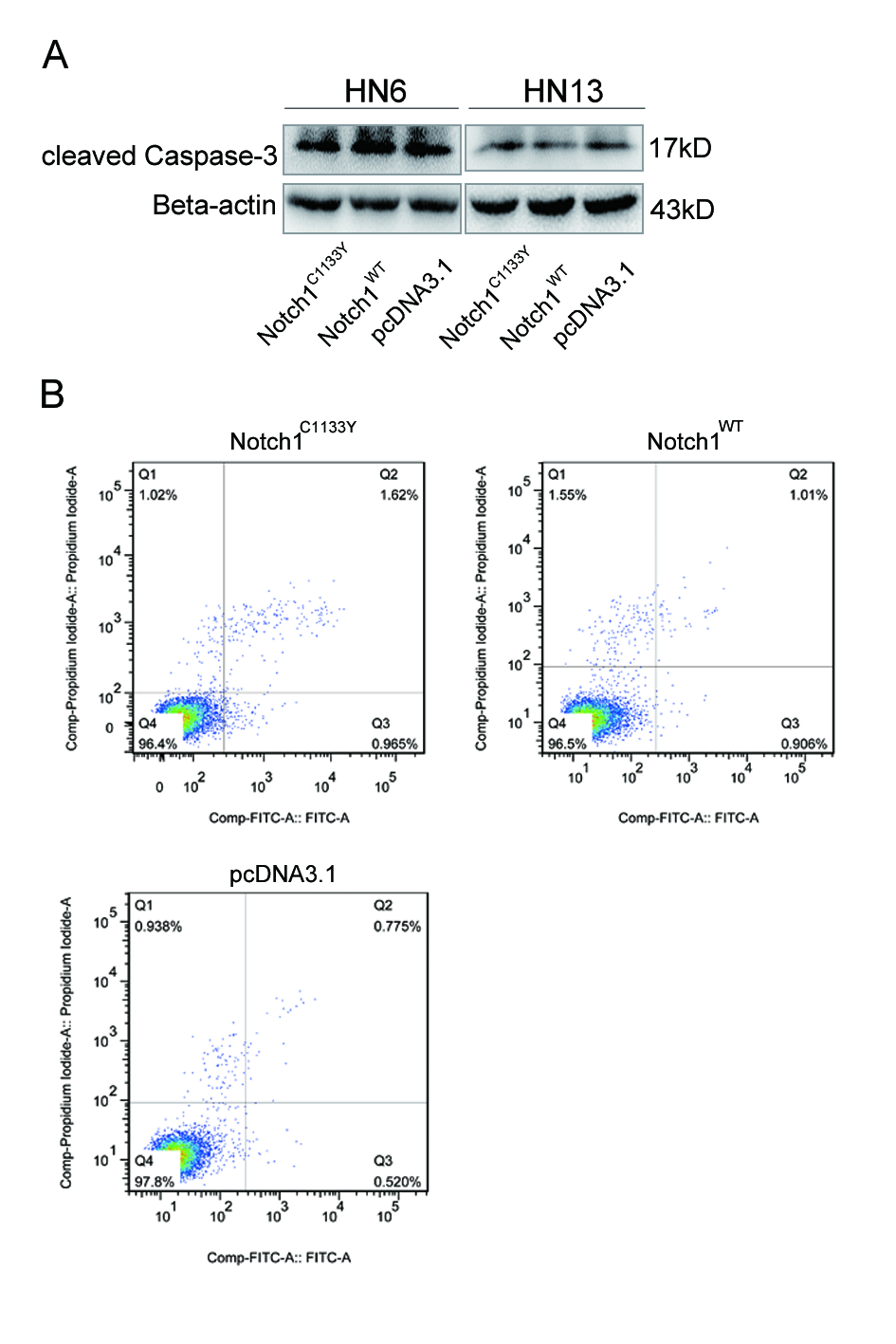

Supplement: Supplementary file 1 — Additional file 1: Figure S1. Cell apoptosis was analyzed in HN6 and HN13 transfected cells. (A) Cleaved Caspase-3 was utilized to detect the cell apoptosis in HN6 and HN13 cells. (B) Flow cytometry was used to determine the early and late stages apoptotic cells. [file 12935_2017_496_MOESM1_ESM.tif]
